# Developing a Framework to Infer Opioid Use Disorder Severity From Clinical Notes to Inform Natural Language Processing Methods: Characterization Study

**DOI:** 10.2196/53366

**Published:** 2024-01-15

**Authors:** Melissa N Poulsen, Philip J Freda, Vanessa Troiani, Danielle L Mowery

**Affiliations:** 1 Department of Population Health Sciences Geisinger Danville, PA United States; 2 Department of Computational Biomedicine Cedars-Sinai Medical Center West Hollywood, CA United States; 3 Department of Autism and Developmental Medicine Geisinger Danville, PA United States; 4 Department of Biostatistics, Epidemiology and Informatics Institute of Biomedical Informatics University of Pennsylvania Philadelphia, PA United States

**Keywords:** annotation, clinical notes, natural language processing, opioid related disorders, opioid use disorder, substance use disorders, adult, adults, opioid, annotation schema, severity score, substance misuse, mental health

## Abstract

**Background:**

Information regarding opioid use disorder (OUD) status and severity is important for patient care. Clinical notes provide valuable information for detecting and characterizing problematic opioid use, necessitating development of natural language processing (NLP) tools, which in turn requires reliably labeled OUD-relevant text and understanding of documentation patterns.

**Objective:**

To inform automated NLP methods, we aimed to develop and evaluate an annotation schema for characterizing OUD and its severity, and to document patterns of OUD-relevant information within clinical notes of heterogeneous patient cohorts.

**Methods:**

We developed an annotation schema to characterize OUD severity based on criteria from the *Diagnostic and Statistical Manual of Mental Disorders, 5th edition*. In total, 2 annotators reviewed clinical notes from key encounters of 100 adult patients with varied evidence of OUD, including patients with and those without chronic pain, with and without medication treatment for OUD, and a control group. We completed annotations at the sentence level. We calculated severity scores based on annotation of note text with 18 classes aligned with criteria for OUD severity and determined positive predictive values for OUD severity.

**Results:**

The annotation schema contained 27 classes. We annotated 1436 sentences from 82 patients; notes of 18 patients (11 of whom were controls) contained no relevant information. Interannotator agreement was above 70% for 11 of 15 batches of reviewed notes. Severity scores for control group patients were all 0. Among noncontrol patients, the mean severity score was 5.1 (SD 3.2), indicating moderate OUD, and the positive predictive value for detecting moderate or severe OUD was 0.71. Progress notes and notes from emergency department and outpatient settings contained the most and greatest diversity of information. Substance misuse and psychiatric classes were most prevalent and highly correlated across note types with high co-occurrence across patients.

**Conclusions:**

Implementation of the annotation schema demonstrated strong potential for inferring OUD severity based on key information in a small set of clinical notes and highlighting where such information is documented. These advancements will facilitate NLP tool development to improve OUD prevention, diagnosis, and treatment.

## Introduction

### Background

Opioid use disorder (OUD), the problematic pattern of opioid use leading to clinically significant distress or impairment, has remained a significant public health burden for over 2 decades in the United States [1]. In 2021, over 9000 opioid-related overdose deaths involved heroin and nearly 17,000 involved a prescription opioid [2]. In 2021, overdose deaths involving any opioid exceeded 80,000, with 88% involving synthetic opioids like fentanyl. In addition to this significant loss of life, approximately 2.7 million people were diagnosed with OUD in 2021 [3], with the economic cost of the US opioid epidemic estimated to be over US $1 trillion in 2017 [4].

OUD is a *Diagnostic and Statistical Manual of Mental Disorders, 5th edition* (DSM-5) clinical diagnosis with varying levels of severity (mild to severe) based on 11 diagnostic criteria endorsed for a given patient. Criteria include items that assess physiological and behavioral symptoms, as well as harmful health and social consequences of opioid use. Access to patients’ OUD status and severity is valuable to patient care. For example, a clinician might use such information to inform their approach for helping manage a patient’s pain (eg, whether to use opioid analgesics or the dose required). Information about OUD severity aids in clinical decision-making regarding appropriate treatment [5], such as determining whether a patient should be referred for psychotherapy versus an inpatient or outpatient treatment facility. OUD severity has also been proposed for use in measurement-based care to track indicators of disease and improved outcomes [6]. For these reasons, accurately identifying OUD status and severity holds crucial importance to the development of patient care strategies and clinical outcome prediction.

### Detecting and Characterizing OUD in Electronic Health Records

Electronic health records (EHRs) include discrete fields containing information such as diagnostic labels or medication orders, as well as fields that contain free text from clinical notes, encounters, and laboratory testing. Numerous algorithms using diagnostic codes have been designed to address problematic opioid use, including identification of patients at risk for prescription opioid misuse [7], OUD prediction [8], nonmedical opioid use detection [9,10], identification of OUD [11], characterization of problematic opioid use [12], and overdose risk prediction [13]. However, the success of such algorithms is limited when diagnostic codes are minimally applied to patient charts. Information about substance use disorders may be missing from EHRs due to a variety of factors, including disjointed care across hospitals, lack of specialty diagnostic expertise, or stigma of a given diagnosis. Thus, OUD can be challenging to isolate within a patient’s medical record [14,15]. Furthermore, most algorithms have been developed in the context of research focusing on patients with prescription opioid misuse or chronic pain. Patients with chronic pain with opioid prescriptions are at increased risk for opioid misuse [16,17], but only focusing on such populations may exclude patient populations with illicit opioid use or otherwise outside of chronic pain treatment. Previous research indicates clinical notes provide a source of rich information that could improve efforts to identify and characterize OUD [12,18,19].

### Importance of Annotation for Developing and Evaluating Natural Language Processing Tools

Natural language processing (NLP) tools have the potential to improve OUD detection and severity characterization, but many NLP frameworks require high quality data with reliably labeled OUD-related information. Common workflows for generating such a data set entail developing a schema (eg, containing classes [entities and events] and attributes [qualifiers]) representing OUD-related information, creating a codebook of instruction for the annotation process, conducting an agreement study to assess schema reliability, and facilitating consensus review of disagreements to generate a reference standard for benchmarking the NLP system [20]. Prior to these steps, it is imperative to understand how and where relevant information is documented in EHRs to inform data extraction and subsequent automation. Generally, this step is not well described in the scientific literature, nor are the documentation patterns of such information well characterized in studies. This step can be critical to informing intelligent search of EHRs and limiting the note types necessary for operationalizing the algorithm, thereby reducing computational effort and potentially improving accuracy. Although prior studies have used NLP to identify problematic opioid use from EHRs [21-26], few have described an annotation process and none have reported documentation patterns for OUD-relevant information within clinical notes.

### Study Objectives

Our long-term goal is to develop an automated NLP method to identify OUD arising from prescription or illicit opioid use and characterize the severity of such use that can be used for future EHR-based studies to drive informatics solutions to improve the prevention, diagnosis, and treatment of OUD through clinical care. Toward this goal, we developed an annotation schema to characterize severity and documented patterns of OUD-relevant information. Using clinical notes of varying type and across encounter settings from a large integrated health system, we annotated OUD symptoms and other relevant information, comparing several heterogenous cohorts—including patients with chronic pain, OUD diagnoses, and receiving medication treatment for OUD—to explore the following questions:

- How accurately can OUD severity be inferred from text from a small number of targeted clinical notes per patient?
- Where and how do clinical teams document OUD-related information, in terms of clinical note types and encounter settings?
- How is OUD-related information documented over time relative to an opioid- or OUD-specific health care encounter?
- What is the frequency of OUD-related concepts and their co-occurrence in clinical notes?

This information, along with the annotation schema developed and described in this study, may be useful in future development of NLP methods to identify and characterize the severity of OUD.

## Methods

### Study Design

We developed an annotation schema to identify patients with OUD stemming from either prescription or illicit opioid use and characterize OUD severity based on DSM-5 criteria. We applied the schema to deidentified clinical notes from patients with varying evidence of OUD and a comparison group with minimal exposure to opioid analgesics.

### Ethical Considerations

The Geisinger Institutional Review Board and University of Pennsylvania reviewed and approved the protocol for this study (2021-0113).

### Study Population

We obtained clinical notes from EHRs of 100 adult patients from Geisinger, a large integrated health system that serves a largely rural area of central and northeast Pennsylvania. We used stratified random sampling to select 20 individuals from each of 5 mutually exclusive groups, stratifying by sex and age categories (18-29, 30-39, 40-49, 50-59, and 60 years and older) to ensure diversity and equal representation in the data set. Study groups were selected from preexisting data sets used in prior studies [11,27,28] to represent various methods of identifying patients with likely or diagnosed OUD from EHRs and a control group (Textbox 1). Further, 2 groups represented chronic pain patients with (at least mild) OUD confirmed through chart review [27] and patient report as to whether their opioid use began with an opioid analgesic prescription (group CP-RX) or not (group CP-nonRX). In total, 2 groups had at least one OUD diagnostic code, but differed as to whether they had an order for medication treatment of OUD such as buprenorphine (group OUD-TX) or not (group OUD-DX). The control group had a single opioid analgesic order in their EHR. Diagnoses and orders used to define study groups occurred within this study’s period of January 2012 to March 2020.

**Inclusion and exclusion criteria for each study group.**

**CP-RX**

- Inclusion criteria:
  Chronic pain: chronic pain was defined as having at least two opioid analgesic prescriptions for nonprogressive musculoskeletal pain.
  Opioid use disorder (OUD; mild, moderate, or severe) confirmed through chart review.
  Opioid use began with opioid analgesic prescription. Based on self-report in a survey question.
- Exclusion criteria:
  Non-European ancestry: individuals with non-European ancestry were excluded because this study’s sample was originally assembled for a genetic study.

**CP-nonRX**

- Inclusion criteria:
  Chronic pain.
  OUD (mild, moderate, or severe) confirmed through chart review.
  Opioid use did not begin with opioid analgesic prescription. Based on self-report in a survey question.
- Exclusion criteria:
  Non-European ancestry: individuals with non-European ancestry were excluded because this study’s sample was originally assembled for a genetic study.

**OUD-DX**

- Inclusion criteria:
  At least one diagnosis code for OUD. International Classification of Disease codes used to define OUD were based on Jennings et al [29].
- Exclusion criteria:
  Chronic pain.
  Order for medications for OUD including buprenorphine, buprenorphine-naloxone, and naltrexone. Geisinger providers did not prescribe methadone for OUD treatment.

**OUD-TX**

- Inclusion criteria:
  At least one diagnosis code for OUD.
  Order for medications for OUD.
- Exclusion criteria:
  Chronic pain.

**Control**

- Inclusion criteria:
  In total, 1 opioid analgesic order.
- Exclusion criteria:
  Chronic pain.
  Diagnosis code for OUD.
  Order for medications for OUD.

### Data Collection

We obtained notes from inpatient, outpatient, and emergency department (ED) encounters. In total, 11 note types were obtained, selected based on clinician input: outpatient clinic notes, progress notes, ancillary progress notes, history and progress (H&P) notes, discharge summaries, ED notes, ED provider notes, ED triage notes, ED support staff notes, communication notes, and lactation notes. We obtained notes for 3 encounter dates per patient: an index date representing either the first observed OUD diagnosis (for OUD-DX and OUD-TX groups and some patients in the CP-RX and CP-nonRX groups) or the most recent opioid analgesic order (for some patients in the CP-RX and CP-nonRX groups and all patients in the control group) and the encounters immediately prior to and following the index date. Multiple notes per patient were obtained for some encounter dates. Notes were deidentified using Philter [30].

### Annotation Schema Development and Procedures

Schema development was based upon pilot work [18]. We revised the pilot schema to map classes onto DSM-5 criteria for characterizing OUD severity and to clarify or eliminate ambiguous concepts. We leveraged the extensible Human Oracle Suite of Tools, an open-source text tool [31] to annotate notes. In total, 2 authors (MNP and PJF) separately reviewed and annotated notes across 15 batches (batched by note type). Annotation was completed at the sentence level, assigning full sentences to one or more relevant classes. After each batch, the 2 reviewers adjudicated discordances through discussion, with other study team members providing input when discordances remained unresolved. We varied the note type annotated in consecutive batches to ensure portability of the schema across note types.

### Severity Score

We calculated a severity score for each patient based on annotations in their notes. Scores used 18 of the annotation schema classes, which mapped onto the DSM-5 criteria for characterizing OUD severity (Multimedia Appendix 1). The “crosswalk” between classes and DSM-5 criteria was based on a systematic chart review process developed by Palumbo et al [27] and adapted by Poulsen et al [11]. Scores ranged from 0 to 11. Severity was categorized based on DSM-5 guidelines (0-1=no OUD; 2-3=mild OUD; 4-5=moderate OUD; >6=severe OUD).

We calculated positive predictive values (PPVs) for detecting moderate or severe OUD among patients with annotations for the 4 study groups with likely or diagnosed OUD, and again for patients categorized into 2 groups based on their index encounter reason (OUD diagnosis or opioid analgesic order). PPVs were calculated as the number of patients with a severity score >4 divided by the total number of patients with annotations. We did not calculate PPVs separately for severity category (mild, moderate, and severe), as there was insufficient information in EHRs on which to base such a comparison. We also calculated PPVs among the full sample of patients (regardless of whether they had annotations), a more conservative quantification of the severity score’s validity that accounts for the lack of OUD-relevant information observed in the reviewed notes.

## Results

### Annotation of Clinical Notes

**Figure 1:**
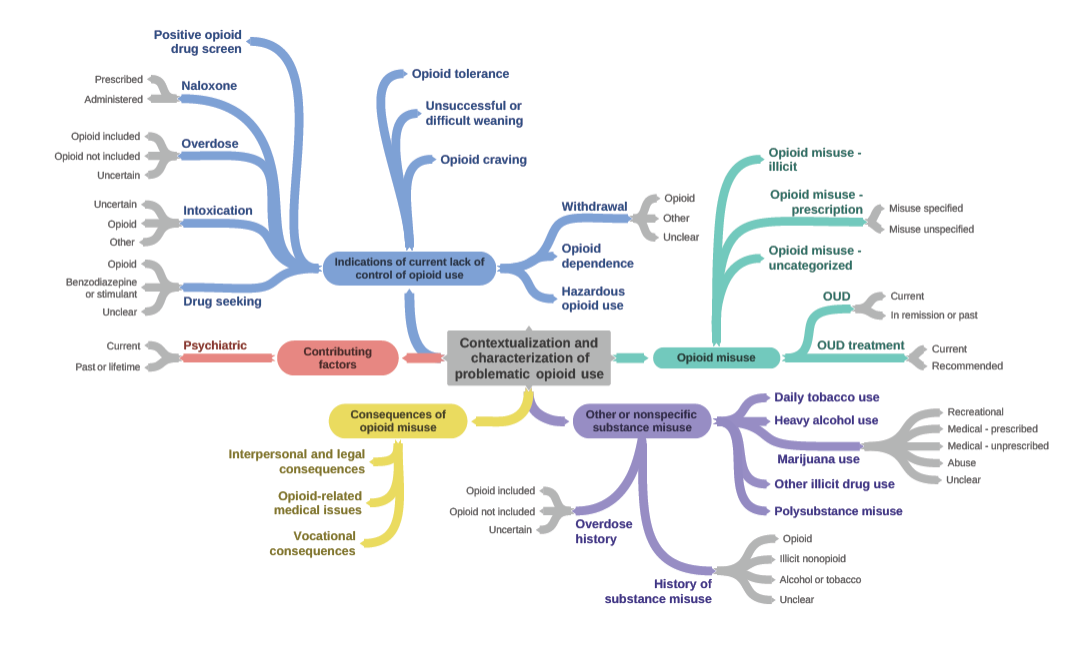
Annotation schema for characterizing OUD severity from clinical note text. OUD: opioid use disorder.

**Figure 2:**
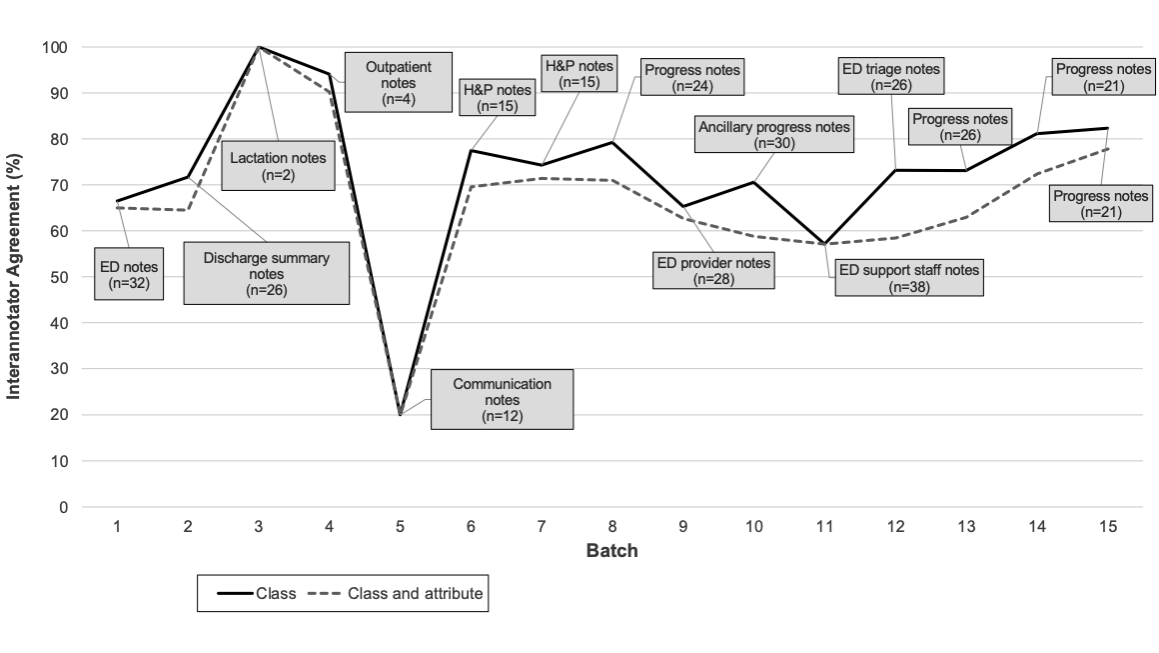
Interannotator agreement across batches of notes with note type and number of notes reviewed per batch. Each batch was a different note type. ED: emergency department; H&P: history and progress.

The annotation schema contained 27 classes, 12 of which included attributes (Figure 1; Multimedia Appendix 2). Interannotator agreement (IAA) for the 2 reviewers ranged from 20% to 100% across the 15 batches of reviewed notes but was above 70% for all except 4 batches (Figure 2). Lower IAA occurred with less frequently annotated classes and for classes with an attribute denoting a historic concept (eg, *psychiatric condition current* vs *historic*; IAA results not shown by class).

From the 100 sampled patients and 320 associated notes, we annotated 1436 sentences within 186 notes from 82 patients over 15 batches. The remaining notes did not yield any annotations (ie, they did not contain text relevant to the classification schema). Most patients without annotations were controls (11/18, 61%) and most had an index encounter based on an opioid analgesic order (16/18, 89%). They also had fewer notes available (mean 1.8, SD 1.1) and no notes were from the inpatient setting.

Among the 4 noncontrol groups, 73 patients had annotations (Table 1). OUD-TX was the only group in which all 20 sampled patients had at least one note with text relevant for annotation and the group accounted for the largest proportion of annotations.

**Table 1:** Counts of patients, notes, and annotated sentences for each study group.

Study group	Count of patients with annotations, n (%)	Index encounter^a^		Count of notes with annotations, n (%)	Count of annotated sentences, n (%)
		OUD^b^ diagnosis, n (%)	Opioid order, n (%)		
CP-RX	16 (20)	8 (50)	8 (50)	29 (16)	170 (12)
CP-nonRX	18 (22)	14 (78)	4 (22)	32 (18)	277 (19)
OUD-DX	19 (23)	19 (100)	0 (0)	50 (27)	363 (25)
OUD-TX	20 (24)	20 (100)	0 (0)	61 (34)	602 (42)
Control	9 (11)	0 (0)	9 (100)	14 (8)	24 (2)

^a^Reason for selecting index encounter, either a diagnosis of OUD or an order for an opioid analgesic.

^b^OUD: opioid use disorder.

### Can Severity of Problematic Opioid Use Be Inferred From a Limited Number of Clinical Notes?

We used annotated classes to calculate severity scores for the 82 patients with annotations. All control group patients had a score of 0. The mean severity score among the 73 patients in noncontrol groups was 5.1 (SD 3.2). The majority (48/73, 66%) had a score >6 (indicating severe OUD), 4 of 73 (5%) had a score of 4-5 (indicating moderate OUD), 2 of 73 (3%) had a score of 2 (indicating mild OUD), and 19 of 73 (26%) had a score of 0-1 (indicating no OUD). Severity scores were highest for the OUD-TX group and lowest for the CP-RX group (Table 2). The mean severity score among these 73 patients was 6.0 (SD 2.7) for those whose index encounter was selected based on an OUD diagnosis and 0.6 (1.7) for those with an index encounter based on an opioid analgesic order.

**Table 2:** Average OUD^a^ scores among 82 patients with annotated sentences.

Study group or index encounter reason^b^	Mean (SD) OUD score	Count of patients by OUD severity					PPVs^c^ for moderate or severe OUD	
		None, n (%)	Mild, n (%)	Moderate, n (%)	Severe, n (%)	Patients with annotations		All sampled patients
CP-RX	3.4 (3.4)	8 (50)	0 (0)	1 (6)	7 (44)	0.50		0.40
CP-nonRX	4.5 (3.9)	7 (39)	0 (0)	1 (6)	10 (56)	0.61		0.55
OUD-DX	5.2 (2.5)	3 (16)	1 (5)	1 (5)	14 (74)	0.79		0.75
OUD-TX	6.8 (2.2)	1 (5)	1 (5)	1 (5)	17 (85)	0.90		0.90
Control	0.0 (0.0)	9 (100)	0 (0)	0 (0)	0 (0)	N/A^d^		N/A
OUD diagnosis	6.0 (2.7)	8 (13)	2 (3)	4 (7)	47 (77)	0.84		0.81
Opioid analgesic order	0.6 (1.7)	11 (92)	0 (0)	0 (0)	1 (1)	0.08		0.03

^a^OUD: opioid use disorder.

^b^Reason for selecting index encounter, either an OUD diagnosis or an order for an opioid analgesic.

^c^PPV: positive predictive value.

^d^N/A: not applicable.

The PPV for detecting moderate or severe OUD among the 73 noncontrol group patients with annotations was 0.71. PPVs were highest for OUD-TX group at 0.90 (Table 2). At 0.84, the PPV for detecting moderate or severe OUD using the notes of patients whose index encounter was selected based on an OUD diagnosis was higher than patients whose index encounter was based on an opioid analgesic order (PPV=0.08).

The mean number of notes per patient that were reviewed and annotated differed slightly by degree of severity (Table 3), but these differences were not statistically significant (1-way ANOVA for mean number of notes reviewed: *F*_3,78_=0.60; *P*=.62; for mean number of notes annotated: *F*_3,56_=1.46; *P*=.24). We observed no consistent patterns in the frequency of note types or encounter types by severity.

*History of substance misuse* was among the most prevalent classes across severity groups and was present in 56-126 (17%-38%) of the 332 notes (Table 3). For those with “no OUD,” the classes *psychiatric condition* and *daily tobacco use* were also common. The class *OUD treatment* automatically led to a classification of “severe OUD” and was present in 36 (16%) of the 226 notes among patients scored as “severe OUD.”

**Table 3:** Note characteristics by OUD^a^ severity among 100 sampled study patients with 332 notes.

Note characteristics			Not annotated		OUD severity						
					None		Mild		Moderate		Severe
**Patients, n**											
			18		28		2		4		48
**Notes reviewed, n**											
			32		98		12		17		226
**Number of notes reviewed per patient, mean (SD)**											
			1.8 (1.2)		3.3 (2.3)		5.0 (0.0)		4.0 (3.0)		3.8 (2.3)
**Total number of notes annotated, n**											
			N/A^b^		90		12		15		214
**Number of notes with annotations per patient, mean (SD)**											
			N/A		2.3 (2.0)		5.0 (0.0)		3.8 (3.0)		3.3 (2.2)
**Note type, n (%)**											
	Progress notes	17 (53)		24 (24)		1 (8)		7 (41)		45 (20)	
	H&P^c^ notes	0 (0)		21 (21)		6 (5)		1 (6)		46 (20)	
	ED^d^ notes	4 (13)		9 (9)		1 (8)		2 (12)		33 (15)	
	ED provider notes	3 (9)		22 (22)		2 (17)		5 (29)		28 (12)	
	Discharge summaries	0 (0)		16 (16)		2 (17)		1 (6)		38 (17)	
	ED triage notes	2 (6)		1 (1)		0 (0)		0 (0)		15 (7)	
	Ancillary progress notes	0 (0)		1 (1)		0 (0)		0 (0)		11 (5)	
	Communication notes	0 (0)		1 (1)		0 (0)		0 (0)		2 (1)	
	OPT^e^ clinic notes	1 (3)		2 (1)		0 (0)		0 (0)		1 (0)	
	ED support staff notes	5 (16)		1 (1)		0 (0)		1 (6)		6 (3)	
	Lactation notes	0 (0)		0 (0)		0 (0)		0 (0)		1 (0)	
**Encounter type, n (%)**											
	OPT	18 (56)		25 (26)		1 (8)		3 (18)		31 (14)	
	ED	14 (44)		33 (34)		5 (42)		10 (59)		97 (43)	
	ED to IPT^f^	0 (0)		21 (21)		6 (50)		4 (24)		67 (30)	
	IPT	0 (0)		18 (18)		0 (0)		0 (0)		31 (14)	
**Most frequent classes (percentage of notes with class)^a^**											
			N/A		History of substance misuse (38%)		History of substance misuse (22%)		Psychiatric condition (22%)		History of substance misuse (18%)
			N/A		Psychiatric condition (31%)		Withdrawal (22%)		History of substance misuse (17%)		OUD treatment (16%)

^a^OUD: opioid use disorder.

^b^N/A: not applicable.

^c^H&P: history and progress.

^d^ED: emergency department.

^e^OPT: outpatient.

^f^IPT: inpatient.

### Where Is OUD-Relevant Information Found in Clinical Notes and How Is it Documented Over Time?

**Figure 3:**
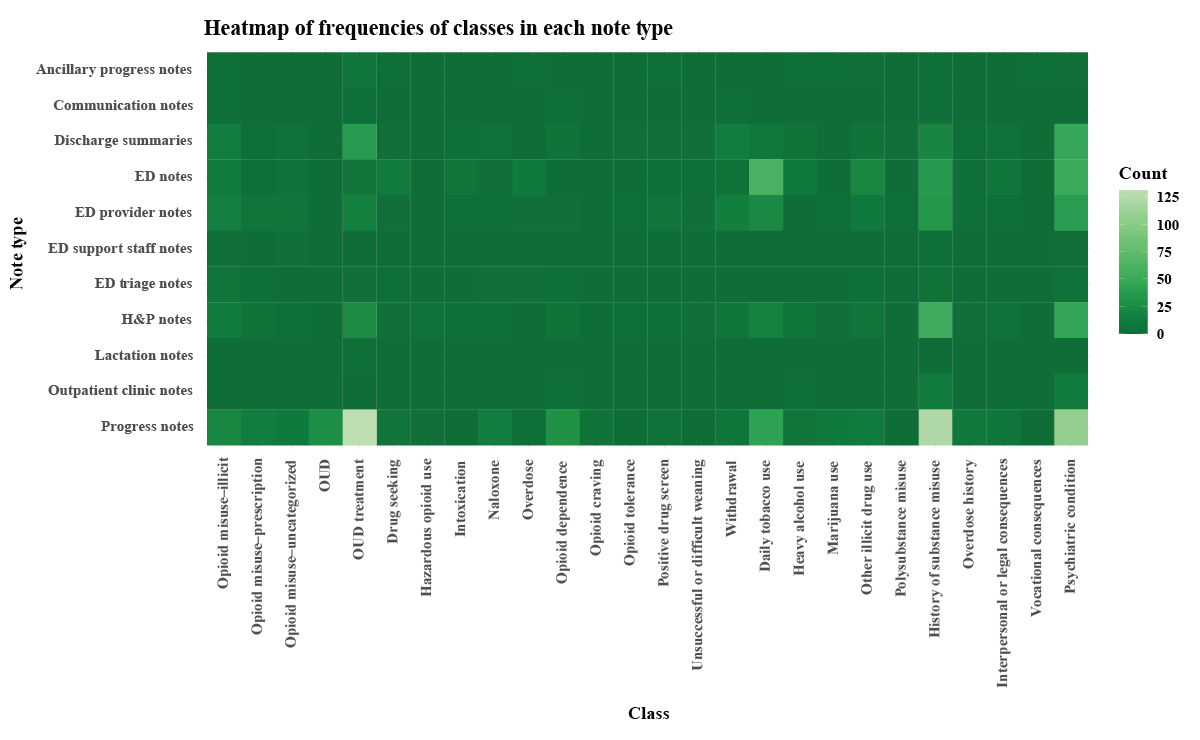
Heatmap of class frequencies by note type. ED: emergency department; H&P: history and progress; OUD: opioid use disorder.

Progress notes, the most common note type in the sample, had the largest total number of annotations, was among the highest yielding note types with an average of 9.3 annotations per note (SD 10.4), had the greatest diversity of classes represented, and was the only note type in which we observed the class *OUD* (signifying a definitive OUD diagnosis; Figure 3; Table 4). However, progress notes had a lower proportion of notes with annotations compared to other note types. H&P notes had the largest proportion of notes with annotations, followed by discharge summaries, ED provider notes, and ED notes (Table 4). These 4 note types also represented a large diversity of classes.

**Table 4:** Count of notes and annotated sentences by note type among the 1436 annotated sentences.

Note type	Number of notes	Number of notes with annotated sentences, n (%)	Counts of notes with annotated sentences by encounter setting (percentage of total)					Number of annotated sentences, n (%)		Mean (SD) number of annotated sentences per note
			ED^a^, n (%)	ED to IPT^b^, n (%)	OPT^c^, n (%)	IPT, n (%)				
Progress notes	92	62 (67)	1 (2)	7 (11)	3 (5)	51 (82)	574 (40)		9.3 (10.4)	
H&P^d^ notes	30	27 (90)	8 (30)	13 (48)	6 (22)	0 (0)	196 (14)		7.3 (5.7)	
ED notes	32	25 (78)	21 (84)	4 (16)	0 (0)	0 (0)	237 (17)		9.5 (9.9)	
ED provider notes	28	23 (82)	18 (78)	5 (22)	0 (0)	0 (0)	177 (12)		7.7 (7.0)	
Discharge summaries	26	23 (88)	5 (22)	10 (43)	8 (35)	0 (0)	172 (12)		7.5 (6.4)	
ED triage notes	26	9 (35)	9 (100)	0 (0)	0 (0)	0 (0)	22 (2)		2.4 (1.7)	
Ancillary progress notes	30	9 (30)	2 (22)	4 (44)	3 (33)	0 (0)	19 (1)		2.1 (1.2)	
Communication notes	12	3 (25)	0 (0)	2 (67)	1 (33)	0 (0)	5 (0)		1.7 (0.6)	
OPT clinic notes	4	2 (50)	0 (0)	0 (0)	0 (0)	2 (100)	26 (2)		13.0 (15.6)	
ED support staff notes	38	2 (5)	1 (50)	1 (50)	0 (0)	0 (0)	7 (0)		3.5 (2.1)	
Lactation notes	2	1 (50)	0 (0)	0 (0)	1 (100)	0 (0)	1 (0)		1.0 (N/A^e^)	

^a^ED: emergency department.

^b^IPT: inpatient.

^c^OPT: outpatient.

^d^H&P: history and progress.

^e^N/A: not applicable.

**Figure 4:**
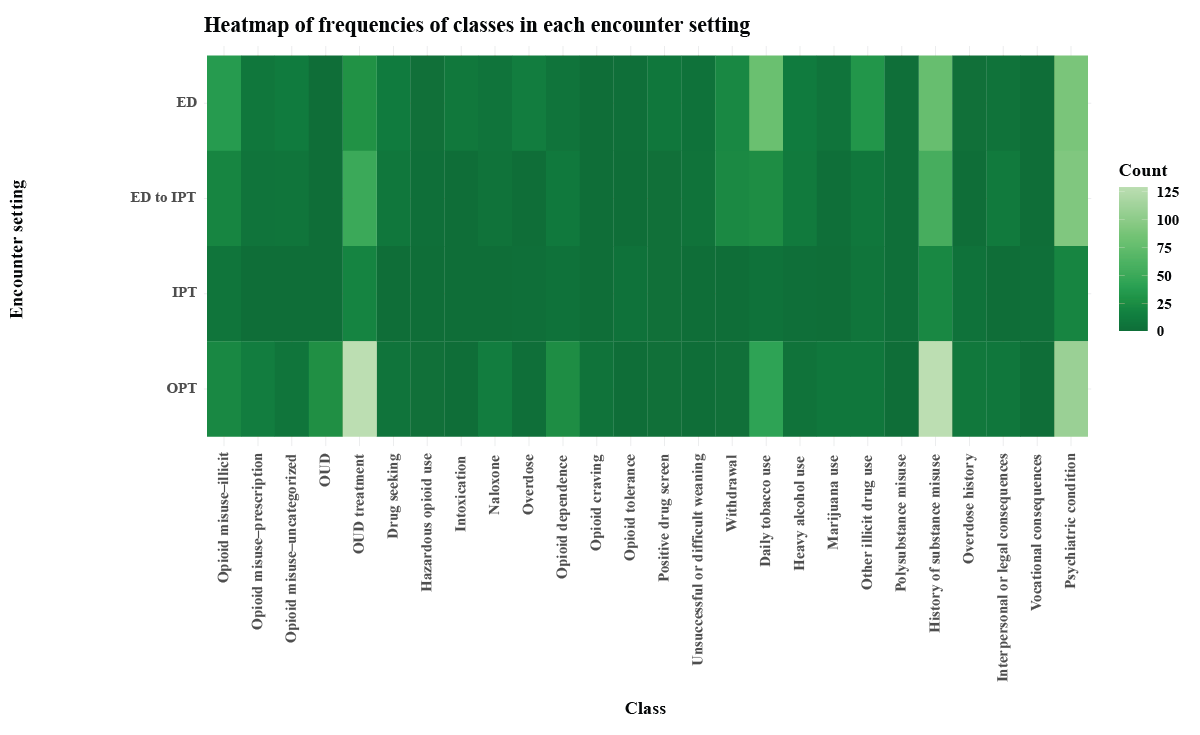
Heatmap of class frequencies by encounter setting. ED: emergency department; IPT: inpatient; OPT: outpatient; OUD: opioid use disorder.

Regarding encounter type, compared to the inpatient setting, notes from ED and outpatient settings had the highest proportions of notes with annotations and a high diversity of classes represented (Figure 4).

Some classes appeared across varied note types, such as *OUD treatment*, *daily tobacco use*, *history of substance misuse*, and *psychiatric condition,* whereas others tended to only appear in a particular note type (eg, *OUD, naloxone, opioid craving, and overdose history* in progress notes; *drug seeking* in ED notes).

**Figure 5:**
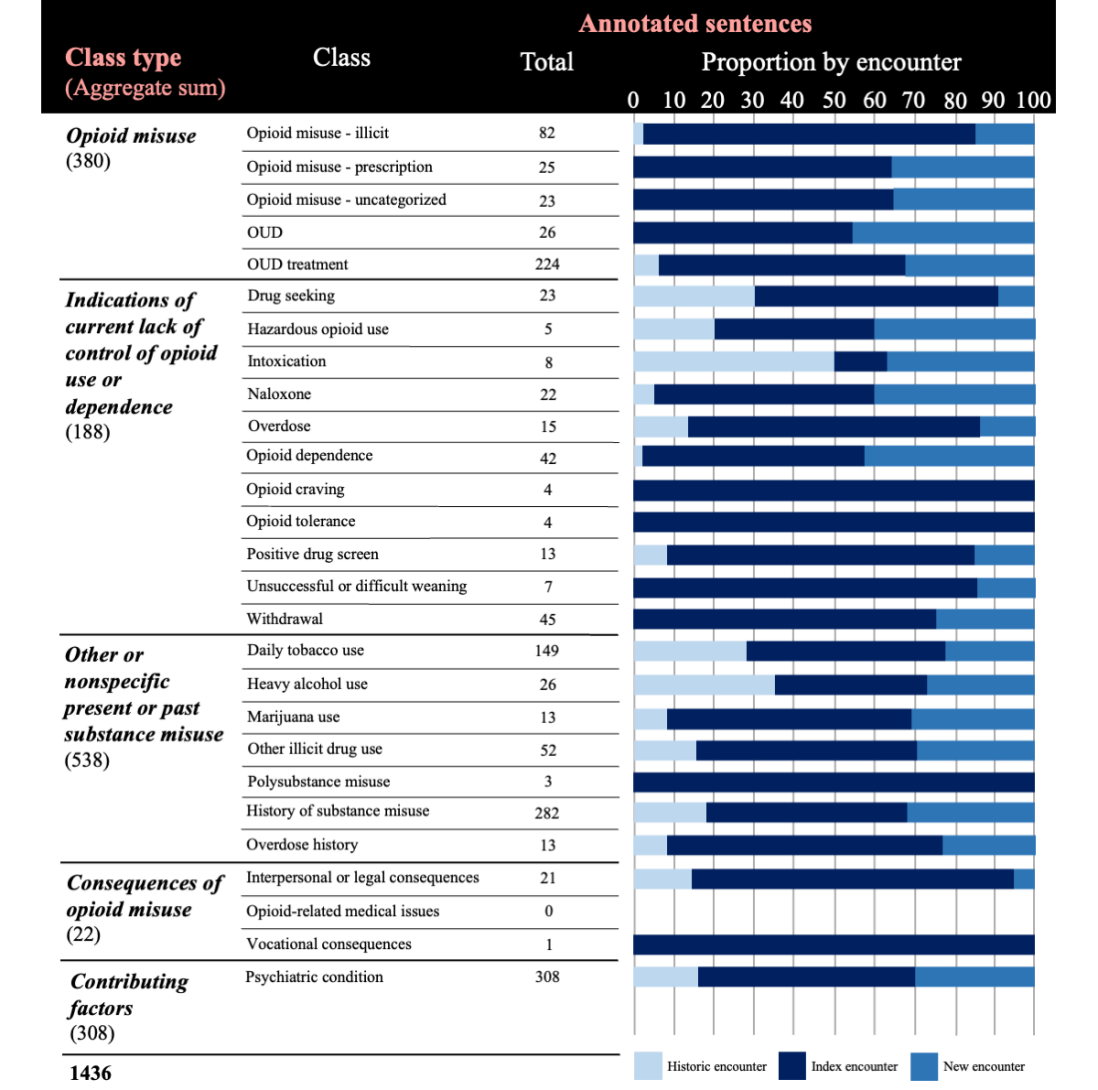
Count of total annotated sentences by class and proportion by encounter order among the 1436 annotated sentences. Note selection was centered around an index encounter, during which a patient first met group inclusion criteria. “Historic” and “new” refer to notes from encounters immediately preceding and proceeding the index encounter, respectively. OUD: opioid use disorder.

The largest proportion of annotations was observed at the index encounter for all classes except *intoxication* (Figure 5). The classes *opioid craving*, *opioid tolerance*, *polysubstance misuse*, and *vocational consequences* were only observed at the index encounter. Annotations were more common in “new” versus “historic” encounters, and several classes were only observed in “new” encounters and not “historic” encounters.

### Which OUD-Related Concepts Are Most Common in Clinical Notes?

The largest number of annotations involved classes representing substance use not specific to opioids, with the most common classes being *history of substance misuse* and *daily tobacco use* (Figure 5)*.* Except for the class *overdose history*, none of these classes contributed to the OUD severity score. Classes representing opioid misuse had the second largest number of annotations, with *OUD treatment* being the most common. *Psychiatric condition*, representing a contributing factor to OUD, was also commonly observed. Few annotations with classes representing consequences of opioid misuse occurred; this category included some of the least common classes including *vocational consequences* and *opioid-related medical issues.* Several of the least commonly assigned classes represented current lack of control of opioid use*.*

### Which OUD-Related Concepts Are Found Together in Clinical Notes?

**Figure 6:**
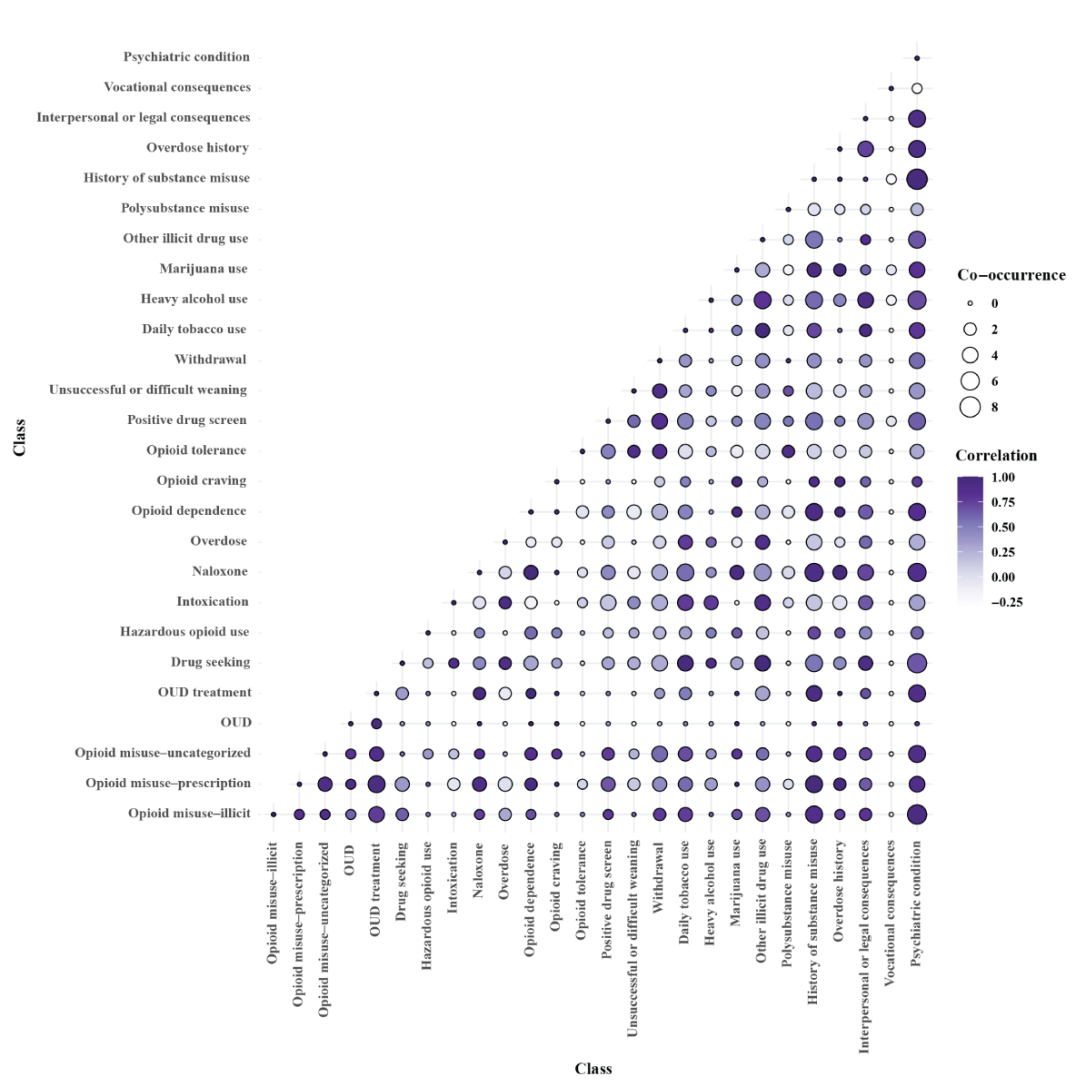
Correlation between and co-occurrence of classes. Color depicts correlations between distributions of class pairs documented across note types, demonstrating how similarly 2 given classes are observed across the 11 note types. High correlation indicates classes are documented with similar frequency distributions across note types; conversely, low correlation indicates classes have different frequency distributions across notes. Bubble sizes depict class frequency co-occurrence at the patient level, showing how frequently 2 given classes are experienced across patients. Higher frequency ranges indicate 2 class pairs are more frequently experienced together by a given patient; conversely lower frequency ranges indicate 2 class pairs are less frequently experienced together. OUD: opioid use disorder.

Several class pairs had highly correlated frequency distributions across note types, indicating similar documentation frequency (Figure 6). *Psychiatric condition*, *history of substance misuse*, *opioid misuse-illicit*, and *opioid misuse-prescription* were highly correlated with many other classes. Conversely, classes with the lowest correlations in their frequency distribution with other classes included *opioid tolerance*, *vocational consequences*, and *polysubstance misuse.*

When evaluated across patients, the classes *psychiatric condition* and *history of substance misuse* also had high frequency of co-occurrence with other classes, as did *other illicit drug use* (Figure 6). Classes with the lowest frequency of co-occurrence with other classes included *opioid tolerance*, *opioid craving*, *vocational consequences*, and *OUD*.

## Discussion

### Principal Results

Through development and evaluation of an annotation schema to characterize OUD severity and documentation of patterns of OUD-relevant information, this study illustrated severity can be inferred from a limited number of clinical notes. Severity, determined by capturing features associated with DSM-5 OUD severity criteria, followed the expected range of scores, with the highest severity observed for patients receiving OUD treatment and the lowest among those with prescriptions for chronic pain. While severity was determined using a range of note types, we found the most relevant information in outpatient notes typically used for acute care and within a subset of note types. The prevalence of schema classes varied widely, providing information regarding the concepts most useful for developing NLP tools.

### Inferring Severity of Problematic Opioid Use

To our knowledge, this is the first study to develop an annotation schema to characterize OUD severity. Most patients received a severity score indicating severe OUD or no OUD. The paucity of scores indicating mild or moderate OUD may be explained by our approach to mapping classes to DSM-5 criteria, the selection of study cohorts, and the limited number of notes reviewed. The “crosswalk” of classes and DSM-5 criteria was based on prior work that included clinician input [11,27], but down weighting specific classes could be justified in future uses and would yield lower severity scores. Additionally, the resulting PPVs for moderate or severe OUD aligned with expectations for this study’s cohorts. Control group individuals all had a severity score of 0, evidence of the specificity of our approach. The OUD-TX group’s PPV of 0.90 is consistent with successful EHR-based algorithms for other conditions [32]. This was expected since these individuals received medication treatment for OUD, which, if documented in the notes we reviewed, would yield a score indicative of severe OUD. The OUD-DX group also had a high PPV, which is unsurprising given their OUD diagnosis. OUD is often underdiagnosed [14,15]; thus, the existence of an OUD diagnosis would be expected to indicate a true disorder, with signs and symptoms likely documented. That said, prior studies have found International Classification of Diseases codes are insufficient for accurately identifying OUD [11,12,14], which may explain why the PPV for OUD-DX was somewhat lower than the OUD-TX group. Both chronic pain groups had moderate PPVs and individuals in these groups were more likely to have a score indicating no OUD. This is likely explained by the index encounter reason—half of the CP-RX group and nearly a quarter of the CP-nonRX group were selected based on an opioid analgesic order. Most individuals whose index encounter was based on an opioid analgesic order had a score indicative of “no OUD.” Although these 2 cohorts had OUD confirmed through a chart review process in prior studies [27,28], the confirmation was only for mild OUD. It is also probable that given the limited number of notes reviewed for this study, additional notes may have yielded information indicative of OUD, which may have occurred later than the 3 selected encounter dates. Based on these findings, we believe that using a limited number of notes is likely sufficient for characterizing OUD severity only in the presence of other confirming information, such as an OUD diagnosis or a medication order for OUD treatment.

### Documentation Patterns of Information Relevant to OUD in Clinical Notes

To inform development of NLP methods, we explored documentation patterns of OUD-related information. We observed the most relevant information—as reflected by the proportion of notes with annotated sentences—were in ambulatory and outpatient settings, as opposed to the inpatient setting. This suggests clinical notes from ED and outpatient settings may yield more relevant information regarding OUD; however, we do not recommend excluding notes from the inpatient setting given that our pilot work demonstrated the ubiquity of OUD-relevant information in hospital discharge summaries [18]. Regarding note type, H&P notes, discharge summaries, ED provider notes, and ED notes yielded the most information pertaining to OUD, and the most diverse information. Finally, relevant information was most often found within notes from the index encounter and the following encounter, demonstrating the importance of selecting notes in reference to relevant structured EHR information and suggesting the sensitivity of OUD severity scores may improve with review of additional notes following the index encounter. The index encounter was defined based on an OUD diagnostic code or the patient’s most recent opioid analgesic order; having such a meaningful anchor may be particularly important for characterizing severity with limited notes. To optimize efficiency, future studies of OUD should consider focusing on the notes likely to yield the most relevant information. However, our findings should be considered preliminary, as we did not surveil all notes, opting for those surrounding the index encounter. Documentation patterns were also likely influenced by the reason for the encounter (eg, whether related to pain management versus medication treatment for OUD). Thus, findings may differ with a review of a patient’s full history of clinical notes.

Opioid misuse, nonopioid substance abuse, and psychiatric classes were the most common annotations in our cohort. The prevalence of opioid misuse classes is not unexpected given that 4 of 5 groups had confirmed OUD. Relatedly, substance use disorders and psychiatric disorders tend to be comorbid with OUD [33]. That said, *history of substance misuse* and *psychiatric condition* were also the most common classes among individuals characterized as having no OUD based on severity criteria. Although nonopioid substance use disorders and psychiatric disorders are risk factors for OUD, they are also commonly comorbid with one another [33]. Indeed, genetic predispositions for substance use disorders, including OUD, and psychiatric disorders can be shared via genetic pleiotropy [34]. The high prevalence and co-occurrence of substance use disorders and psychiatric disorders was also observed across note types and patients. Taken together, these results highlight the importance of substance use and psychiatric classes for developing OUD-related NLP tools.

Absence of certain concepts is also informative for NLP tool development. Consistent with previous work [18,27], some classes relevant to OUD severity were rarely observed, including those representing consequences of opioid misuse and lack of control of opioid use. Reviewing a larger set of notes in an expanded patient sample could yield more frequent documentation of some classes, but some classes may represent concepts not traditionally recorded by clinicians (eg, *vocational consequences*). Documentation by clinicians of additional concepts such as the vocational, social, legal, and medical consequences patients face due to opioid use could be useful for patient care, particularly given the potential utility of such information in characterizing OUD severity. Future work to develop NLP frameworks related to OUD should consider that information captured in clinical notes may change over time with secular changes that occur in the drug landscape, clinical practices and documentation, and patients’ care-seeking behaviors.

### Limitations

This study used clinical notes from an integrated health system, allowing for review of multiple note types across encounter settings. Findings may be different in health systems in which information is not integrated across settings; for example, opioid analgesic orders may be missing and OUD treatment siloed. Although we evaluated cohorts with varying evidence of opioid misuse, inclusion of other patient cohorts may yield different findings. In particular, because the cohorts were originally assembled for a genomic study, the CP-RX and CP-nonRX study groups included individuals only of European ancestry, limiting the generalizability of findings to other racial groups. Replication studies are necessary to understand whether study findings generalize to other settings and patient populations.

### Conclusions

Understanding how and where OUD-relevant information is captured in EHRs is essential to informing development of NLP tools to identify and characterize the severity of OUD. We developed an annotation schema to determine OUD severity and highlighted document patterns of OUD-relevant information in clinical notes, which may be informative for future NLP frameworks related to OUD. Findings suggest OUD-relevant information is more prevalent in a subset of note types in ambulatory and outpatient settings—particularly H&P notes, discharge summaries, ED provider notes, and ED notes—and that certain information relevant to OUD may only be captured in certain note types or may be infrequently documented. Furthermore, when reviewing a limited number of notes, having a meaningful anchor such as an OUD diagnostic code or recent opioid analgesic order is important for characterizing OUD severity. Findings also demonstrate the potential for inferring severity of OUD from key information contained in a limited number of clinical notes, paving the way for development of informatics solutions to improve the prevention, diagnosis, and treatment of OUD through clinical care.

## Appendix

Multimedia Appendix 1 Crosswalk of class or attribute combinations and Diagnostic and Statistical Manual of Mental Disorders, 5th edition (DSM-5) criteria for opioid use disorder (OUD).

Multimedia Appendix 2 Classes with brief definitions, example annotated sentences, and the count of annotated sentences by class or attribute.

## Abbreviations

DSM-5: Diagnostic and Statistical Manual of Mental Disorders, 5th edition
ED: emergency department
EHR: electronic health record
H&P: history and progress
IAA: interannotator agreement
NLP: natural language processing
OUD: opioid use disorder
PPV: positive predictive value
