# Supplementary material for: Developing a Framework to Infer Opioid Use Disorder Severity From Clinical Notes to Inform Natural Language Processing Methods: Characterization Study
Source: JMIR Ment Health. 2024 Jan 15;11:e53366. doi: 10.2196/53366 (PMC10825772; doi:10.2196/53366)
Supplement: Multimedia Appendix 1 [file mental_v11i1e53366_app1.docx]

**Multimedia Appendix 1.** Crosswalk of class/attribute combinations and DSM-5 criteria for OUD.

| **Class/attribute combinations** | **DSM-5 criteria^1^ for OUD severity** | | | | | | | | | | |
| --- | --- | --- | --- | --- | --- | --- | --- | --- | --- | --- | --- |
|  | **1** | **2** | **3** | **4** | **5** | **6** | **7** | **8** | **9** | **10** | **11** |
| Positive drug screen/Opioid | X | X | X | X |  |  |  |  | X | X |  |
| Drug seeking/Opioid | X | X | X | X |  |  |  |  |  | X |  |
| Opioid misuse–illicit  Opioid misuse–prescription  Opioid misuse–uncategorized | X |  | X | X |  |  |  |  | X | X |  |
| Overdose/Opioid or Unclear  Naloxone | X |  |  |  |  |  |  | X | X |  |  |
| Intoxication/Opioid | X |  |  |  |  |  |  | X |  |  |  |
| Withdrawal/Opioid | X |  |  |  |  |  |  |  |  |  | X |
| OUD treatment/Current | X | X | X | X |  |  |  |  |  | X | X |
| Unsuccessful or difficult weaning |  | X |  |  |  |  |  |  |  | X | X |
| Opioid craving |  | X |  | X |  |  |  |  |  |  |  |
| Opioid tolerance  Opioid dependence |  |  |  |  |  |  |  |  |  | X |  |
| OUD treatment/Recommended | X | X | X | X |  |  |  |  |  | X | X |
| Vocational consequences |  |  |  |  | X |  | X |  | X |  |  |
| Opioid-related medical issues |  |  |  |  |  | X | X |  | X |  |  |
| Interpersonal and legal consequences |  |  |  |  |  | X | X |  | X |  |  |
| Hazardous opioid use |  |  |  |  |  |  |  | X |  |  |  |
| ^1^DSM-5 criteria for OUD include: 1—More or longer use of opioids than intended; 2—Unsuccessful effort to cut down or control opioid use; 3—Excessive time spent obtaining or using opioids or recovering their effects; 4—Craving; 5—Recurrent opioid use resulting in failure of work/school/home obligations; 6—Continued opioid use despite social/interpersonal problems from opioids; 7—Reduced social/occupational/recreational activities due to opioid use; 8—Continued opioid use when physically hazardous; 9—Continued use despite physical or psychological problems likely caused by opioids; 10—Tolerance; 11—Withdrawal | | | | | | | | | | | |
