# Supplementary material for: Developing a Framework to Infer Opioid Use Disorder Severity From Clinical Notes to Inform Natural Language Processing Methods: Characterization Study
Source: JMIR Ment Health. 2024 Jan 15;11:e53366. doi: 10.2196/53366 (PMC10825772; doi:10.2196/53366)
Supplement: Multimedia Appendix 2 [file mental_v11i1e53366_app2.docx]

**Multimedia Appendix 2.** Classes with brief definitions, example annotated sentences, and the count of annotated sentences by class or attribute.

| **Class** | **Definition** | **Example annotated sentence** | **Count of annotated sentences by attribute** |
| --- | --- | --- | --- |
| ***Opioid misuse*** | | |  |
| Opioid misuse–  illicit | Recent illicit opioid misuse (e.g., heroin) | “Pt reports last use of heroin yesterday” | 82 (no attributes) |
| Opioid misuse–prescription | Recent prescription opioid misuse | “Pt taking Percocet he gets from a friend” | True: 21  Unclear: 4 |
| Opioid misuse–uncategorized | Non-specific mentions of opioid/narcotic abuse | “Final diagnoses: Opioid abuse” | 23 (no attributes) |
| OUD | Specific mention of patient having OUD | “Assessment: opioid use disorder” | Current: 20  In remission/past: 6 |
| OUD treatment | OUD treatment mentioned during encounter | “On suboxone”  “Referral for substance use disorder counseling” | Current: 143  Past:31  Recommended: 50 |
| ***Indications of current lack of control of opioid use/opioid dependence*** | | |  |
| Drug seeking | Indications of drug-seeking behavior | “Pt keeps asking for pain medications” | Opioid: 12  Other: 3  Unclear: 8 |
| Hazardous opioid use | Opioid use in situations physically hazardous to the patient or others | “Has used needles and shared them” | 5 (no attributes) |
| Intoxication | Patient arrived at encounter intoxicated | “Pt came in for altered mental status due to being drunk” | Opioid: 0  Other: 6  Uncertain: 2 |
| Naloxone | Patient given naloxone | “Education regarding opioid overdose, Naloxone prescription provided” | Administered: 5  Prescribed: 16  Other: 1 |
| Overdose | Overdose a concern in current encounter | “Presenting problem: OD” | Opioid included: 4  Opioid not included: 0  Uncertain 11: |
| Opioid dependence | Specific mention of opioid “dependence” | “Narcotic dependency” | 42 (no attributes) |
| Opioid craving | Mention of craving for opioids | “She reported strong cravings at present” | 4 (no attributes) |
| Opioid tolerance | Mention of tolerance to opioids | “Pt has developed narcotic tolerance” | 4 (no attributes) |
| Positive drug screen | Positive opioid drug screening results | “Oxycodone positive” | 13 (no attributes) |
| Unsuccessful or difficult weaning | Patient has had unsuccessful efforts to reduce opioid use | “Pt attempted to quit on her own but was unsuccessful” | 7 (no attributes) |
| Withdrawal | Mentions of withdrawal/withdrawal symptoms | “Pt was found to have opioid withdrawals” | Opioid: 32  Other: 4  Unclear: 9 |
| ***Other or non-specific present or past substance misuse*** | | |  |
| Daily tobacco use | Recent^b^ daily use of tobacco | “Tobacco use: Yes Packs/Day: 0.25” | 149 (no attributes) |
| Heavy alcohol use | Recent heavy use of alcohol | “Alcohol abuse” | 26 (no attributes) |
| Marijuana use | Recent marijuana use | “Cannabis: Regular daily user x 1 years” | Recreational: 1  Medical, prescribed: 0  Medical, unprescribed: 0  Abuse: 4  Unclear: 8 |
| Other illicit drug use | Recent illicit drug use, opioids not specified | “Injected cocaine a few days ago” | 52 (no attributes) |
| Polysubstance misuse | Specific mentions of “polysubstance abuse” when substances are not named | “Secondary diagnosis: polysubstance abuse” | 3 (no attributes) |
| History of substance misuse | History of drug misuse | “He was on cocaine for many years” | Alcohol/tobacco: 86  Illicit non-opioid: 34  Opioid: 89  Unclear: 73 |
| Overdose history | Indication that patient has overdosed in the past | “Pt reports an overdose history of 4 times” | Opioid included: 4  Opioid not included: 0  Uncertain: 11 |
| ***Consequences of opioid misuse*** | | |  |
| Interpersonal or legal consequences | Opioid use impacting interpersonal life (relationships) or leading to legal issues | “Pt in legal trouble for stealing to buy drugs” | 21 (no attributes) |
| Opioid-related medical issues | Physical or psychological problems exacerbated by opioid use | n/a | 0 (no attributes) |
| Vocational consequences | Opioid use impacting patient’s work, school, or employments status | “Hasn’t been able to keep employment” | 1 (no attributes) |
| ***Contributing factors*** | | |  |
| Psychiatric condition | Patient has an acute mental health/psychiatric condition | “Patient being admitted for suicidal ideation”  “Past medical history: anxiety” | Current: 176  Past/lifetime: 132 |
